# Case Report: An Unusual Case of Pulmonary Metastatic Adenocarcinoma From Low-Grade Appendiceal Mucinous Neoplasms

**DOI:** 10.3389/fonc.2022.906344

**Published:** 2022-07-13

**Authors:** Xin-Yu Zhao, Chun-Qiang Li, Shi-Yao Zhang, Gang Liu

**Affiliations:** Department of General Surgery, Tianjin Medical University General Hospital, Tianjin, China

**Keywords:** case report, appendiceal tumor, low-grade appendiceal mucinous neoplasm, pulmonary metastases, next-generation sequencing

## Abstract

**Background:**

Low-grade appendiceal mucinous neoplasms (LAMNs) are indolent tumors with low-grade cytology. Although peritoneal dissemination is common due to tumor rupture and mucinous deposits on the visceral peritoneal surface, distant involvement, such as lung, is rarely seen due to lack of invasiveness.

**Case Presentation:**

A 70-year-old woman presented to the hospital due to continuously elevated carcinoembryonic antigen (CEA) levels for 10 months without any symptoms. PET/CT revealed two lesions located in the left lung and appendix. The postoperative pathology results revealed pulmonary mucinous adenocarcinoma and LAMN. Then we performed next-generation sequencing (NGS) to clarify the relationship between the two tumors. The sequencing result showed that both tumors harbored the common tumor mutations, KRAS (p.G12D), GNAS (p.R201H), and BRAF (p.R735Q), which indicated that the pulmonary tumor was a metastasis of LAMN.

**Conclusion:**

This case is unusual in that the primary LAMN and the pulmonary metastasis are present at the time of diagnosis. This study reported the first pulmonary metastasis from LAMN verified by NGS.

## Introduction

Low-grade appendiceal mucinous neoplasms (LAMNs) are typically well-differentiated and indolent tumors. Differing from appendiceal mucinous adenocarcinoma, LAMNs feature a “push” pattern of growth instead of invasiveness, and distant metastasis rarely appears. Pseudomyxoma peritonei (PP) is a rare disease, characterized by a “gelatinous abdomen” or mucinous ascites from intra-abdominal neoplastic mucin-secreting cells proliferating on the peritoneal surface (1). The majority is secondary to mucinous neoplasms of the appendix. Appendiceal mucinous neoplasms cause cystic dilatation of the appendix due to the accumulation of copious amounts of gelatinous material. This usually leads to appendicular rupture, resulting in dissemination throughout the peritoneal cavity in the form of gelatinous deposits (2, 3). The mucus-producing cells continue to proliferate on the peritoneal surface, and mucinous fluid gradually fills the peritoneal cavity, resulting in the characteristic “gelatinous abdomen” (4). The patient usually remains asymptomatic for a long time before the diagnosis is performed (5). The most common clinical presentation includes an increase in abdominal girth (3). Intestinal obstruction by bowel adhesion is also a main symptom in the late stage of the disease.

Although pleuropulmonary metastatic tumor from LAMNs is rare because of the benign growth pattern, several case reports of intrathoracic extension of mucinous material have been published. Reviewing previous studies, we found that all pulmonary metastases of LAMNs were confirmed by pathology. How to distinguish between primary mucinous adenocarcinoma and metastatic mucinous adenocarcinoma is greatly influenced by pathologists. At present, next-generation sequencing (NGS) has been fully applied to tumor genetics. Through the mutation information between tumors, it can accurately infer the relationship between two tumors. In this study, we first applied NGS technology to diagnose the pulmonary metastatic adenocarcinoma from LAMNs.

## Case Presentation

**Figure 1:**
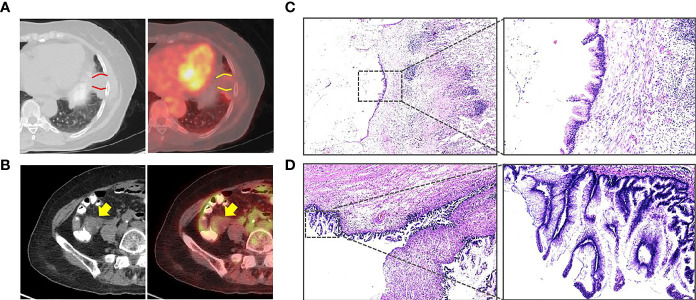
**(A)** PET/CT showing that an effusion can be seen in the oblique fissure of the left lung, and a nodular thickening was also seen near the pleura in the lower lobe of the left lung (between the two curves, standard uptake value (SUV) = 2.08). **(B)** PET/CT showing that a cystic mass can be seen in the ileocecal area (arrow, SUV = 2.28). **(C)** The pulmonary tumor revealed mucinous adenocarcinoma (H&E). **(D)** The postoperative pathology revealed a low-grade appendiceal mucinous neoplasm (H&E).

A 70-year-old woman presented to the outpatient service in our hospital because of continuously elevated carcinoembryonic antigen (CEA) levels detected during a routine examination (from 9.43 to 28.02 ng/ml) for 10 months without any symptoms. During this period, no sign was found on abdominal CT and colonoscopy. To clarify the source of CEA, PET/CT was arranged for this patient. The examination revealed effusion in the oblique fissure of the left lung, and nodular thickening (standard uptake value (SUV) = 2.08) was also present near the pleura in the lower lobe of the left lung (**Figure 1A**). The lesion size was 7 × 3 × 1 cm^3^. In addition, a cystic mass (**Figure 1B**) was located in the ileocecal area, and the SUV at the edge of the mass was 2.28. The patient consulted a thoracic surgeon. The thoracic surgeon organized a multidisciplinary team (MDT) to formulate plans for treatment, including our treatment team. After discussion, the patient and her family were consulted, and the final treatment plan was determined: two surgeries were successively arranged for the pulmonary tumor and ileocecal tumor.

The pathologic analysis of the pulmonary tumor revealed mucinous neoplasms (**Figure 1C**). Immunohistochemistry disclosed positivity for CK7 and CK20 and negativity for napsin A, TTF1, and SATB2, which confirmed that the pulmonary tumor was metastatic adenocarcinoma. Therefore, we decided to perform right hemicolectomy for her. Because the patient suffered a pulmonary infection after the thoracic surgery, the abdominal surgery was not performed until 3 months. However, postoperative pathology revealed a LAMN but not mucinous adenocarcinoma (**Figure 1D**).

**Figure 2:**
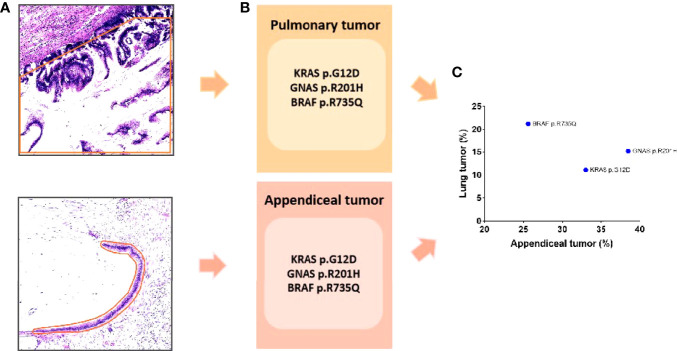
**(A)** The tumor was accurately sampled from a section under the microscope (the orange line indicates the sampling range). **(B)** The sequencing results show that both tumors had the same mutation: KRAS (p.G12D), GNAS (p.R201H), and BRAF (p.R735Q). **(C)** The variant allele frequency is shown in scatter diagram.

After the H&E staining of two tumors was reviewed, it was found that both tumors harbored low-grade cytology and a “push” growth pattern. To further clarify the relationship between tumors, NGS was performed for this patient. Tumor cells from sections of the tumors were accurately collected under a microscope for NGS because of the small number of tumor cells in the mucinous neoplasm (**Figure 2A**). The results illustrated that both tumors carried the same tumor-specific mutations, namely, KRAS (p.G12D), GNAS (p.R201H), and BRAF (p.R735Q) (**Figures 2B, C**), which indicated that the pulmonary tumor was a metastasis of LAMN.

## Discussion

There have been only a few cases of pulmonary involvement from primary LAMN, and most cases describe pulmonary involvement long after the initial diagnosis. The earliest case was reported by Berge (6), who described a case of pulmonary metastases in a patient with an appendiceal mucocele. Later, Mortman et al. (7) reported three patients with PP and low-grade mucinous appendiceal tumors that later developed pulmonary nodules after cytoreduction with peritoneal disease recurrence. After that, several cases have been reported. However, the mechanism of pulmonary metastasis in LAMN is still unclear. Geisinger et al. (8) reviewed 38 patients with pleuropulmonary involvement of appendiceal mucinous neoplasm. In this study, the mechanisms of pleuropulmonary involvement in most cases were related to diaphragm defects. However, in rare cases, the tumor may directly pass through an intact diaphragm *via* the lymphovascular spaces or *via* direct invasion. The metastases in these cases are often accompanied by low-grade cytological features (8–10), including our case report. In appendiceal mucinous neoplasm, the tumor with low-grade cytological features is almost lacking in invasion. Even if it spreads to the abdominal cavity, it also lacks the ability of infiltrative invasion into subjacent tissues (4). Therefore, the mechanism of pulmonary metastasis needs to be further explored.

To date, almost all cases of pleuropulmonary involvement in LAMN were verified by pathology or imaging. KRAS and GNAS are the most commonly mutated genes in patients with LAMNs (11). KRAS mutation is considered to occur early in the tumorigenesis of LAMNs. Mutated GNAS usually increases intracellular cyclic adenosine monophosphate levels, and it is considered to play a direct role in the prominent mucin production (12). However, BRAF mutation is rare in LAMNs (4). Mutated BRAF is a major driver of gene alteration in cancers of multiple tissue origins. However, the mutation in BRAF in this case was not located in the primary domains. Therefore, the effect of mutated BRAF in this case needs further investigation.

In conclusion, this case is unusual in that the primary appendiceal tumor is low-grade cytological atypia and that the pulmonary metastasis is present at the time of diagnosis. This study reported the first pulmonary metastasis of LAMN verified by NGS. Our study provided a new diagnostic strategy for further investigating distant metastasis of LAMNs.

## Data Availability Statement

The original contributions presented in the study are included in the article/supplementary material. Further inquiries can be directed to the corresponding author.

## Ethics Statement

This study was reviewed and approved by The Ethical Committee of Tianjin Medical University General Hospital. Written informed consent was obtained from the individual for the publication of any potentially identifiable images or data included in this article.

## Author Contributions

X-YZ, C-QL, and S-YZ wrote the manuscript and were assistants during the surgery. GL was the chief operating surgeon. All authors contributed to the article and approved the submitted version.

## Conflict of Interest

The authors declare that the research was conducted in the absence of any commercial or financial relationships that could be construed as a potential conflict of interest.

## Publisher’s Note

All claims expressed in this article are solely those of the authors and do not necessarily represent those of their affiliated organizations, or those of the publisher, the editors and the reviewers. Any product that may be evaluated in this article, or claim that may be made by its manufacturer, is not guaranteed or endorsed by the publisher.
